# Evaluating the implementation of rapid diagnostic tests in a malaria elimination setting

**DOI:** 10.1186/s40249-020-00702-6

**Published:** 2020-07-08

**Authors:** Di Liang, Jia-Jie Jin, Wei-Ming Wang, Yuan-Yuan Cao, Guo-Ding Zhu, Hua-Yun Zhou, Jun Cao, Jia-Yan Huang

**Affiliations:** 1 Key Lab of Health Technology Assessment, National Health Committee, Shanghai, China; 2 School of Public Health, Fudan University, Shanghai, China; 3 Key Laboratory of National Health Commission on Parasitic Disease Control and Prevention, Key Laboratory of Jiangsu Province on Parasite and Vector Control Technology, Jiangsu Institute of Parasitic Diseases, Wuxi, China; 4 Center for Global Health, School of Public Health, Nanjing Medical University, Nanjing, China; 5 Public Health Research Center, Jiangnan University, Wuxi, China

**Keywords:** Malaria, Rapid diagnostic tests, Access to care, Pretest-posttest control group design

## Abstract

**Background:**

It was recommended that malaria rapid diagnostic tests (RDTs) should be available in all epidemiological situations. But evidence was limited on the implementation of RDTs and its effectiveness in malaria elimination settings. This study examined the implementation of RDTs and how it affected the diagnosis of imported malaria patients in Jiangsu Province, China.

**Methods:**

To scale up RDTs, this study developed an intervention package with four major elements covering the supply of RDT test, the training on RDTs, the monitoring and management of RDT use, and the advocacy of RDTs. By using a pretest-posttest control group design, we implemented the interventions in 4 cities in Jiangsu Province with the rest nine cities as controlled areas, from January 2017 to January 2018. Difference-in-Difference approach was used to evaluate the impact of the scale-up of RDTs on the identification of malaria cases. Three binary outcome measures were included to indicate delayed malaria diagnosis, malaria cases with confirmed malaria diagnosis at township-level institutions, and severe malaria cases, respectively. Linear probability regression was performed with time and group fixed effects and the interaction term between time and group.

**Results:**

Intervention areas received sufficient RDT test supply, regular professional training programs, monthly tracking and management of RDT supply and use, and health education to targeted population. The implementation of interventions was associated with 10.8% (*P* = 0.021) fewer patients with delayed diagnosis. But intervention areas did not see a higher likelihood of having confirmed diagnosis from township-level institutions (coefficient = -0.038, *P* = 0.185) or reduced severe malaria cases (coef. = 0.040, *P* = 0.592).

**Conclusions:**

The comprehensive package of RDT implementation in this study is promising in scaling up RDT use and improving access to care among malaria patients, especially in malaria elimination settings.

## Background

In 2015, the World Health Assembly set ambitious goals to dramatically lower the global malaria burden by 2030 in a new Global Technical Strategy for Malaria 2016–2030 [1]. A key milestone for 2020 is to achieve zero indigenous cases of malaria in at least 10 countries that had the disease in 2015. In 2016, the World Health Organization (WHO) identified 21 countries with the potential to achieve this goal. Supported by WHO and other partners, these 21 malaria-eliminating countries formed the E-2020 initiative to eliminate malaria by 2020. In 2018, seven out of the 21 countries reported zero indigenous cases [2]. China is part of the E-2020 initiative. China’s National Malaria Elimination Program, which was launched in 2010, also aimed at eliminating malaria by 2020 [3, 4]. China has recorded zero indigenous cases of malaria since August 2016 and will soon be eligible to be certified as malaria free by WHO [5].

Despite the impressive progress made in China, achieving elimination and maintaining zero indigenous cases is not without its challenges. On the one hand, imported malaria is a constant threat. On the other hand, it is increasingly difficult to maintain a group of technicians skilled at malaria microscopy at the grassroot level [6]. Microscopy was the only tool routinely available for malaria diagnosis, and experienced lab technicians are highly concentrated in tertiary hospitals and Centers for Diseases Control (CDCs). But febrile patients usually visit township health centres first after symptom onset. The mismatch of diagnostic capacities and needs may prevent malaria patients from receiving timely diagnosis and treatments, which may threat the elimination status of malaria [4, 6].

It is widely recommended that rapid diagnostic tests (RDTs) should be readily available in places where technicians are not skilled in malaria microscopy, in all epidemiological situations [7]. Malaria RDTs are lateral flow immuno-chromatographic antigen-detection tests to detect specific antigens produced by malaria parasites in the blood (commonly obtained from a finger-prick) of infected individuals [8]. When a parasite antigen binds to the dye-labeled antibody, the resultant complex is captured by a band of bound antibody on the strip, forming a visible line in the results window. RDTs can detect only one species (*Plasmodium falciparum* [*P. f.*]) or multiple species (*P. vivax*, *P. malariae* and *P. ovale*) [9–11]. RDTs have much shorter testing time, user-friendly testing procedure, and similar accuracy compared to microscopy [10, 12–15]. Thus, RDT is particularly appropriate in improving the accessibility and efficiency of malaria testing for febrile patients at the grassroot level [16]. However, many clinical, economic, logistical, and social concerns remain about the roll-out of RDTs [17–19]. Though guidelines had been available [18], evidence was limited on the implementation of RDTs and the impact of RDT expansion on healthcare utilization among malaria patients in malaria-eliminating countries [20–25].

We implemented interventions to promote the use of RDTs and evaluated whether RDTs improved the diagnostic efficiency for malaria, in Jiangsu Province, China. With 80 million people, Jiangsu Province was ready for roll out RDTs for several reasons. First, Jiangsu Province was the in the elimination phase, and RDTs are likely to be most cost-effective in areas with low malaria transmission [21]. No indigenous case was reported in Jiangsu Province since 2012. But the reported malaria cases in Jiangsu Province ranked among the top of provinces in China. The yearly number of imported malaria cases in Jiangsu Province ranged from 198 to 405 in 2012–2016. All malaria cases in Jiangsu Province were imported, and the majority of them were *P. f.* infection. Jiangsu Province had a large group of overseas migrant workers in Sub-Saharan Africa. Movement of overseas migrant workers contributed to the seasonal fluctuation of malaria epidemic. Second, Jiangsu Province faced the challenge to maintaining the capacity of malaria microscopy at the grassroot level, and the efficiency of malaria diagnosis using microscopy remained low. Third, the costs of RDTs were covered by the government. The scale-up of RDTs would not have a financial impact on patients and providers.

Before the interventions, Jiangsu Province had several challenges implementing RDTs. First, RDTs were not routinely available in township health centres, as RDT supply was limited. Even when available, RDTs were not integrated into the workflow of malaria diagnosis. Providers at the grassroot level were not familiar with RDTs due to lack of training [26]. Furthermore, no supporting and quality control strategies of RDTs were provided to these healthcare institutions. In this study, we hypothesized that interventions to scale up RDTs would improve access to malaria diagnosis: 1) reduce delayed confirmed diagnosis, 2) increase malaria diagnosis at the grassroot level (in this study, grassroot level institutions are operationalized as township-level institutions), and 3) reduce severe cases of malaria.

## Methods

### Intervention design

This study is approved by the IRB of Jiangsu Institute of Parasitic Diseases (IRB00004221/FWA00008405). Interventions were implemented in four out of 13 cities in Jiangsu Province: Changzhou, Huai’an, Taizhou, and Yangzhou, from January 2017 to January 2018. These four cities were purposely selected because they had relatively heavy burden of malaria, particularly imported malaria. In 2012–2017, these four cities reported 42.7% of malaria cases in Jiangsu Province. Other nine cities in Jiangsu Province were controls and would receive interventions after the impact evaluation.

After consulting key informants (29 healthcare providers and laboratory technicians in the four intervention cities), an intervention package for optimizing RDTs use and management was developed tailored to the existing malaria control system in Jiangsu Province. The package includes the following measures:

- Sufficient RDT supply

During the intervention, free RDTs (*P. f.* lactate dehydrogenase (LDH) and malaria pan-LDH) were provided by Jiangsu Institute of Parasitic Diseases in intervention cities. In each intervention city, RDTs were allocated to CDCs and hospitals at the county/district level and healthcare institutions at the township level based on population sizes and the number of overseas migrant workers.

b) Regular training programs for health professionals

In intervention cities, physicians, laboratory technicians, and malaria control professionals at CDCs received two sessions of standardized training on malaria diagnosis and treatment. The training covered epidemiology, diagnosis (with a focus on RDT), and treatment of malaria. In the second sessions of training, implementation progress and feedback from onsite supervision were also delivered to trainees.

c) Process management of RDT supply and use

All healthcare institutions and CDCs receiving RDTs were required to submit monthly tracking tables of the allocation, delivery, receipt, and clinical use of RDTs. The monthly tracking system of RDT supply and use served as the tool of process management during intervention implementation.

d) Enhanced health education for target populations.

Our target populations included healthcare providers, laboratory technicians, and residents who had ever travelled or worked in areas of high malaria transmission. Health education was delivered through printed materials, traditional media, and new media. For instance, we developed a malaria knowledge quiz on Weibo (one of the top social network platforms in China) to raise awareness for malaria [27].

We also applied onsite supervision and quality control measures to ensure the interventions were implemented according to the protocol. The study team supervised the intervention implementation by visiting county-level hospitals and CDCs and township health centres in intervention cities every two months. During onsite supervision, malaria control experts and CDC officers from Jiangsu Institute of Parasitic Diseases reviewed the reserved samples from both RDT-positive and negative patients, documented RDT supply, use, and test results data, and collected comments and suggestions for the implementation. Certain patients were reached by telephone to verify the information documented by healthcare institutions and professionals.

### Study design

We used the malaria surveillance data from 2014 to 2017 in Jiangsu Province. This data source covered the information of healthcare utilization and diagnosis (e.g., the time interval between first visit and confirmed diagnosis, the institution where the patient received confirmed diagnosis) for each malaria case reported in Jiangsu Province. The diagnosis of each malaria case was double-checked by the local CDC. Healthcare utilization information was obtained from the epidemiological survey that was conducted for every case reported by the local CDC.

By adopting a pretest-posttest control group design, we evaluated whether the interventions improved patient access to prompt malaria diagnosis and treatment. We included the following outcome measures: a binary variable indicating whether a patient experienced delayed malaria diagnosis (confirmed diagnosis over 4 days after first visit), a binary variable indicating whether a patient confirmed his or her malaria diagnosis at township health centers, and a binary variable indicating a severe malaria case. Severe malaria cases can reflect delays in parasitic malaria diagnosis, [28] as standardized antimalarial treatment is guaranteed for all patients with confirmed malaria diagnosis in Jiangsu Province.

Difference-in-Difference (DID) method was used to evaluate the impact of the scale-up of RDTs. DID removes biases in post-intervention period comparisons between the intervention and control areas that could be the result from pre-intervention differences between those areas, as well as biases from comparisons over time in the intervention areas that could be the result of trends due to other causes of the outcome [29]. DID was implemented as an interaction term between time (the year of 2017) and intervention group (Changzhou, Huai’an, Taizhou, and Yangzhou) dummy variables in a regression model.

### Statistical analysis

All statistical analyses were performed using Stata software (StataCorp, College Station, USA), version 14.2. Simple proportions were used for most analyses. *χ*^2^ test was used to compare the three outcomes between the intervention and control areas in 2014–2016 and 2017. To facilitate the interpretation of the results, linear probability regression was performed for DID method with time (before and after the implementation) and group (intervention and control areas) fixed effects and their interaction term (the product of time and group indicators). Results were reported using the Standards for Reporting Implementation Studies (StaRI).

## Results

### Process evaluation

- Sufficient RDT supply.

During the intervention, Jiangsu Institute of Parasitic Diseases provided 8000 free RDTs to Changzhou and 10 000 free RDTs to Huai’an, Taizhou, and Yangzhou. By December 2017, among all free RDTs, 73.1% (27 778/38 000) were received by county- and township-level institutions (Table 1).

**Table 1:** Number of RDTs received by county- and township-level institutions by city, 2017

	Changzhou	Huai’an	Yangzhou	Taizhou	Total
201704	3575	-	4135	3824	11 534
201705	324	5621	525	851	7321
201706	75	744	1550	50	2419
201707	136	700	880	126	1842
201708	411	375	175	495	1456
201709	165	375	95	625	1260
201710	58	343	25	75	501
201711	0	175	375	183	733
201712	50	637	0	25	712
Total	4794	8970	7760	6254	27 778

Note: Changzhou, Yangzhou, and Taizhou started to receive free RDTs from April 2017, while Huai’an started to receive free RDTs from May 2017

*RDTs* Rapid Diagnostic Tests

b) Regular training programs for health professionals.

Two training sessions were delivered in April–May 2017 and January 2018. In total, 2372 trainees participated the two training sessions.

c) Process management of RDT supply and use.

By December 2017, the four intervention cities used 23 609 RDTs (Table 2). The use of RDTs peaked during June–September, as migrant workers usually came back home in summer to stay with their children.

**Table 2:** Number of RDTs used and cases reported by city, 2017

	Changzhou		Huai’an		Yangzhou		Taizhou	
	RDT use	case	RDT use	case	RDT use	case	RDT use	case
201704	167	2	-	-	101	1	234	3
201705	468	6	729	5	852	1	801	1
201706	661	4	883	1	693	2	1183	1
201707	759	3	975	4	1152	0	1523	2
201708	764	1	900	2	731	2	1380	5
201709	684	0	794	2	721	2	1133	2
201710	491	1	653	0	434	2	909	0
201711	205	1	415	3	478	2	551	3
201712	186	0	375	1	165	1	459	1
Total	4385	18	5724	18	5327	13	8173	18

Note: Changzhou, Yangzhou, and Taizhou started to receive free RDTs from April 2017, while Huai’an started to receive free RDTs from May 2017

*RDTs* Rapid Diagnostic Tests

d) Enhanced health education to target populations.

For healthcare providers and laboratory technicians, we provided daily used office supplies (e.g. mouse pads, desk calendars, mugs) with printed messages on malaria control. For overseas migrant workers, we leveraged local television platform, display screens in major public places, advertisements before films, and leaflets with essential malaria disease and healthcare services information to educate them essential malaria prevention knowledge and the importance of timely healthcare after suspicious malaria symptoms occur. A total of 916 users took the Weibo quiz on the 10th China Malaria Day (April 26th, 2017). Reader-friendly articles and posters were regularly posted though Jiangsu Institute of Parasitic Diseases official WeChat (one of the top social network platforms in China).

### Effectiveness evaluation

The intervention and control areas did not differ significantly (11.8% vs 15.8%, *P* = 0.059) in the percentage of patients experiencing delayed malaria diagnosis (receiving confirmed diagnosis over 4 days after first visit) in 2014–2016. But the intervention areas had significantly lower percentage of patients with delayed treatment (6.0% vs 20.7%, *P* = 0.003) in 2017 (Table 3). In linear probability regression, the interaction term between time and intervention group dummy variables was significant (coefficient = -0.108, *P* = 0.021), while the time (coef. = 0.048, *P* = 0.181) and intervention group (coef. = -0.039, *P* = 0.065) dummy variables did not have significant coefficients (Table 3). In other words, intervention implementation in the intervention group was negatively associated with 10.8% more delayed diagnosis among malaria patients.

**Table 3:** Descriptive results of major outcomes in intervention and control areas

(%)		Before intervention					After intervention	
		2014	2015	2016	2014–2016 average		2017	Change in 2017 compared to 2014–2016 average
Confirmed diagnosis over 4 days after 1st visit	Intervention areas	18.1	8.1	9.3	11.8		6.0	-5.8
	Control areas	16.4	15.5	15.6	15.8		20.7	+ 4.9
Receiving confirmed diagnosis at THCs	Intervention areas	2.3	6.5	5.4	4.7		3.6	-1.1
	Control areas	2.7	2.3	0.6	1.9		4.5	+ 2.6
Severe malaria	Intervention areas	5.3	5.9	0.8	4.0		1.2	-3.2
	Control areas	6.0	2.3	3.9	4.1		3.2	-0.9

*THCs* Township health centers

The intervention and control areas differed significantly in the percentage of malaria cases receiving confirmed diagnosis at township health centers in 2014–2016 (4.7% vs 1.9%, *P* = 0.009) and 2017 (3.6% vs 4.5%, *P* = 0.728) (Table 3). In linear probability regression, the year 2017 (coef. = 0.026, *P* = 0.137) dummy variable and the interaction term (coef. = -0.038, *P* = 0.185) were all not statistically significant, while intervention group (coef. = 0.028, *P* = 0.011) was associated with 2.8% higher likelihood of receiving confirmed diagnosis at the township level (Table 4).

**Table 4:** Regression results of major outcomes

	Confirmed diagnosis over 4 days after 1st visit		Receiving confirmed diagnosis at THCs		Severe malaria	
	coef. (95% *CI*)	*P*-value	coef. (95% *CI*)	*P*-value	coef. (95% *CI*)	*P*-value
Intervention city	-0.039 (-0.080–0.0025)	0.065	0.028 (0.0064–0.050)	0.011	-0.00079 (-0.021–0.019)	0.938
Intervention period	0.048 (-0.022–0.12)	0.181	0.026 (-0.0084–0.061)	0.137	-0.011 (-0.042–0.020)	0.484
Intervention city × Intervention period	-0.108 (-0.20−0.016)	0.021	-0.038 (-0.094–0.018)	0.185	-0.020 (-0.061–0.022)	0.354

Note: Linear probability regression model was used

*Coef.* coefficients of regression models. *CI* Confidence interval

In sensitivity analysis, among all confirmed malaria cases in 2017, 48.8% was reported by county and township institutions in intervention areas, while 46.5% was reported by county and township institutions in control areas. In linear probability regression, the intervention group (coef. = -0.016, *P* = 0.594) dummy variable and the interaction term (coef. = 0.040, *P* = 0.592) were all not statistically significant, while the year 2017 (coef. = -0.116, *P* = 0.010) had 11.6% lower likelihood of receiving confirmed diagnosis at township health centers.

The intervention and control areas did not differ significantly in the percentage of patients with severe malaria in 2014–2016 (4.0% vs 4.1%, *P* = 0.762) as well as in 2017 (1.2% vs 3.2%, *P* = 0.337) (Table 3) In linear probability regression, the time (coef. = -0.011, *P* = 0.484) and intervention group (coef. = -0.00079, *P* = 0.938) dummy variables as well as their interaction term (coef. = -0.020, *P* = 0.354) were not statistically significant (Table 4).

## Discussion

This study developed a comprehensive package of RDT implementation with four major elements including sufficient RDT test supply, regular health professional training programs, monthly tracking and management of RDT supply and use, and enhanced health education to targeted population. We found that the implementation of interventions was associated with reduced delayed diagnosis among malaria patients. This is consistent with findings obtained from both countries working on malaria control and those pursuing sustainable malaria elimination [17, 28, 30–32]. But the implementation of interventions was not associated with a higher likelihood of having confirmed diagnosis from township-level institutions or reduced severe malaria cases.

Multiple explanations might account for the lack of associations between the scale-up of RDTs and confirmed diagnosis at the grassroot level or occurrence of severe malaria. Regarding confirmed diagnosis at the grassroot level, the percentage of patients getting confirmed diagnosis at township-level institutions was affected by these institutions’ diagnosis capacities as well as residents’ trust on them. Although RDTs might be promising to enhance the diagnosis capacities of township health centres, professionals at township health centres were less likely to self-report to be capable of performing standard RDT operations compared to professionals at county-level hospitals and CDCs [26]. Unfortunately, the actual rates of performing standard RDT operations were unknown across institutions. Notably, in the sensitivity analysis, we found no association between interventions and confirmed diagnosis from county or township-level institutions as well. One explanation might be our study period might be too short to observe significant changes for both township-level and county-level institutions. Also, it took time to build trust and change care-seeking behaviours among community members. Moreover, the lack of associations between the scale-up of RDTs and severe malaria might be due to the lack of power. Severe malaria cases were relatively rare. In 2017, only one severe malaria case was reported in intervention areas, while five were reported in control areas.

This implementation research highlighted that supporting strategies and quality control measures are the key to successfully scale-up of RDTs. Our study provided a package of intervention measures including sufficient RDT test supply, regular monitoring and management, training, and advocacy activities. Our measures were aligned with RDT implementation guidelines which emphasized components including planning and coordination, communication and social mobilization, training, monitoring and quality control [33]. These interventions have been implemented in all cities in Jiangsu Province since 2018. Our study contributed to the existent literature by providing empirical evidence on the importance of these supporting strategies and quality control measures.

This study has several limitations and should be interpreted with caution. First, intervention areas were not selected by randomization. In this real-world implementation study, the selection intervention areas had to account for feasibility issues. Thus, we did not assume exchangeability between intervention and control areas. Instead, we used DID to evaluate the effectiveness of interventions, assuming that intervention and control areas had similar trends in outcomes if without interventions. Second, we had a relatively short study period, which might limit the power to detect significant findings and long-term changes after the implementation. As mentioned above, interventions reported here have been implemented in both intervention and control cities in Jiangsu Province since 2018. As a result, we could not extend the post-intervention study period to 2018 and later.

## Conclusions

The comprehensive package of RDT implementation in this study was promising in scale up RDT use and improve access to malaria diagnosis. This implementation research provided experiences for malaria elimination settings and areas where malaria transmission is low. In these settings, the goals were to effectively manage the transmission risk of imported malaria cases and the maintenance of working achievements towards malaria elimination. Our findings emphasized the importance of strengthening malaria diagnosis capabilities at the grassroot level to achieve these goals.

## Data Availability

The data will be available upon requested.

## Abbreviations

CDCs: Centers for Diseases Control
*CI*: Confidence interval
DID: Difference-in-Difference
LDH: lactate dehydrogenase
RDTs: Rapid Diagnostic Tests
StaRI: Standards for Reporting Implementation Studies
THCs: Township health centers.
WHO: World Health Organization
